# Prevalence and predictors of active and passive smoking in Saudi Arabia: A survey among attendees of primary healthcare centers in Riyadh

**DOI:** 10.18332/tid/202214

**Published:** 2025-03-14

**Authors:** Seema Nasser, Mamdouh M. Shubair, Amani Alharthy, Faris Fattani, Badr F. Al-Khateeb, Aljohrah I. Aldubikhi, Awad Alshahrani, Lubna Alnaim, Saif Iqbal, Fatmah Othman, Ashraf A. El-Metwally

**Affiliations:** 1 Department of Nursing, College of Nursing, King Saud bin Abdulaziz University for Health Sciences, Riyadh, Saudi Arabia; 2 King Abdullah International Medical Research Center, Saudi Arabia Ministry of National Guard Health Affairs, Riyadh, Saudi Arabia; 3 School of Health Sciences, University of Northern British Columbia, Prince George, Canada; 4 Department of Health Science, College of Health and Rehabilitation Sciences, Princess Nourah Bint Abdulrahman University, Riyadh, Saudi Arabia; 5 Riyadh Second Health Cluster, Riyadh, Saudi Arabia; 6 Department of Family Medicine, King Abdulaziz Medical City, Saudi Arabia Ministry of National Guard Health Affairs, Riyadh, Saudi Arabia; 7 College of Public Health and Health Informatics, King Saud bin Abdulaziz University for Health Sciences, Riyadh, Saudi Arabia; 8 College of Health Sciences, Saudi Electronic University, Riyadh, Saudi Arabia; 9 Department of Medicine, King Abdulaziz Medical City, Saudi Arabia Ministry of National Guard Health Affairs, Riyadh, Saudi Arabia; 10 College of Medicine, King Saud bin Abdulaziz University for Health Sciences, Riyadh, Saudi Arabia; 11 Clinical Nutrition Department, College of Applied Medical Sciences, King Saud Bin Abdulaziz University for Health Sciences, Riyadh, Saudi Arabia; 12 College of Medicine, Alfaisal University, Riyadh, Saudi Arabia

**Keywords:** active smoking, passive smoking, secondhand smoke, sociodemographic factors, Saudi Arabia

## Abstract

**INTRODUCTION:**

Smoking remains a leading cause of preventable diseases worldwide, including cancer, heart disease, and respiratory disorders. In Saudi Arabia, the prevalence of smoking has been increasing, particularly among men and adolescents. However, limited research has focused on the prevalence and predictors of active and passive smoking in the region, particularly within the adult population. Understanding the sociodemographic and health-related factors that influence smoking behaviors can inform tobacco control strategies. The aim of the study is to investigate the prevalence and predictors of active and passive smoking among adults attending primary healthcare centers in Riyadh, Saudi Arabia.

**METHODS:**

This cross-sectional study was conducted in Riyadh, Saudi Arabia, between March and July 2023, targeting patients aged ≥18 years who visited primary healthcare centers. Multistage cluster sampling was used to select 48 healthcare centers from an initial list of 103 centers. Participants were recruited from the waiting areas, and a total of 14239 individuals completed an electronic questionnaire. The questionnaire assessed sociodemographic information, smoking behavior, and health conditions. Data were analyzed using SPSS version 26.0 for Windows, with Descriptive statistics and multivariable logistic regression analyses to identify factors associated with active and passive smoking. Statistical significance was set at p<0.05.

**RESULTS:**

The prevalence of active smoking was 17.3% and passive smoking was 16.5% among the participants. The multivariate logistic regression analysis identified several key predictors for both active and passive smoking. Male gender, larger household size, and lower income were significant factors for active smoking, with individuals in larger households (3–5 members) (AOR=1.48; 95% CI: 1.22–1.79) and those earning between 10000–19000 Saudi Arabian Riyals (AOR=0.56; 95% CI: 0.41–0.75) showing higher odds. Perceived health status also played a role, with those reporting good health (AOR=2.96; 95% CI: 1.68–5.25) having higher odds of smoking. Males were more likely to engage in active smoking compared to females (AOR=2.59; 95% CI: 2.23–3.02). For passive smoking, similar trends were observed, with larger households (AOR=2.27; 95% CI: 1.387–3.721) and male gender (AOR=2.59; 95% CI: 2.23–3.02) being significant predictors.

**CONCLUSIONS:**

The study highlights male gender, larger household size, lower income, and better perceived health status as significant predictors for both active and passive smoking behaviors in Riyadh, Saudi Arabia. These factors should be prioritized in public health strategies aimed at reducing tobacco exposure and promoting cessation. Further research is needed to explore the broader societal factors contributing to smoking behavior and exposure to secondhand smoke in the country.

## INTRODUCTION

Smoking is a significant risk factor for numerous health conditions and represents a major global public health issue, contributing to over 8 million deaths annually^1^. Smoking not only significantly increases the risk of serious health conditions for the smoker, such as lung cancer, heart disease, infectious diseases, and respiratory illnesses, but also poses substantial risks to others through secondhand smoke^2^. Secondhand smoke, or passive smoking, refers to the involuntary inhalation of smoke from another person’s cigarette. Secondhand smoke contains >7000 chemicals, many of which are toxic and carcinogenic. Each year, about 1.2 million of the 8 million smoking-related deaths occur among non-smokers who are exposed to secondhand smoke^1^.

In 2013, 12.2% of adults in Saudi Arabia were reported as smokers, with nearly 75% of those individuals smoking around 15 cigarettes daily^3^. A 2018 survey on tobacco use found that smoking prevalence had risen to 21.4% among adults, marking a 9.2% increase since 2013^4^. This upward trend suggests that exposure to secondhand smoke may have similarly increased. A 2021 study in Saudi Arabia further revealed that smoking rates are notably higher among men than women^5^. Smoking habits typically begin in adolescence, with an estimated 90% of new smokers starting before the age of 18 years^6^. Various factors have been linked to adolescent smoking, including older age, male gender^5^, parental smoking^7^, friends who smoke^7,8^, socioeconomic status^7^, and family issues^9^. Conversely, older smokers often have more established habits and may smoke for reasons different from younger individuals^10^. Additionally, research has shown smoking prevalence is highest among divorced individuals and lowest among those who are married^11,12^.

While there are some studies on smoking predictors and prevalence in Saudi Arabia, the body of research remains limited, particularly regarding adolescent smoking^13^. Moreover, studies on adult smoking predictors are even rarer. Investigating the factors that predict smoking and identifying the groups in which smoking is prevalent is essential for implementing more effective preventive measures to reduce smoking with-in the population^13,14^. In terms of passive smoking, there is a lack of comprehensive research on its prevalence and risk factors, especially concerning the adult population. While some studies focus on adolescents, they are often constrained by limited age ranges and specific demographic groups^14^. Furthermore, much of the existing research on both active and passive smoking is restricted by small sample sizes and highly selective populations, making it difficult to generalize the findings to the broader Saudi population^15^. To address this gap, the current study aims to estimate the prevalence of both active and passive smoking and identify the predictors associated with smoking in Saudi Arabia.

## METHODS

### Study design and eligibility criteria

Between March and July 2023, a cross-sectional study was undertaken in Riyadh, Saudi Arabia, targeting individuals attending primary healthcare centers. To be eligible, participants needed to be at least 18 years old and registered as patients at any of the selected centers. Healthcare professionals and staff employed at these centers were excluded from the study.

### Study sample and sampling technique

The study employed a multistage cluster sampling approach, where 48 primary healthcare centers were randomly chosen from an original list of 103 centers across Riyadh. In the first stage, 48 centers were selected at random, followed by a random selection of patients within each chosen center. The data for this study were collected by a team of trained data collectors who were recruited through a thorough process. These data collectors underwent prior training on research ethics and data collection procedures to ensure consistency and compliance with ethical standards. This transparency in methodology helps to maintain the integrity of the data collected and enhances the credibility of the study. Data collectors approached patients and family members aged ≥18 years in the waiting areas of these centers at different times of the day. Variations in time during the data collection process can impact the results obtained. By approaching patients and family members at different times of the day, researchers can gather a more comprehensive understanding of the population being studied and potentially capture any time-based variations in their responses. This approach allows for a more well-rounded analysis of the data collected, considering any potential fluctuations that may occur throughout the day. Ultimately, 14239 individuals completed the survey, forming the study’s analytical sample.

### Pilot testing and validation of study questionnaire

An electronic questionnaire was developed and initially pilot-tested in Hail, Saudi Arabia, outside the study’s main location. The questionnaire, adapted from English-language sources, was first translated into Arabic and then back-translated into English to ensure accuracy. Prior to administering it to the 14239 patients, a pilot test with 100 patients in Hail was conducted to evaluate question clarity and comprehensiveness, leading to necessary refinement. Additionally, insights from a focus group of 20 key informants in Hail contributed to further improvements. After these adjustments, the updated questionnaire was tested again with a different group of 100 patients to evaluate test-retest reliability, yielding a coefficient of 0.83. Experts also assessed the questionnaire’s face validity, confirming it as satisfactory. The face validation and test-retest reliability evaluations were conducted in January 2023, prior to the commencement of the actual data collection in March 2023. The questions were translated to Arabic and subsequently back translated into English to assure language accuracy, as they were exclusively available in English.

### Definitions

Active smoking refers to the act of inhaling tobacco smoke directly from a cigarette, cigar, or pipe^16^. The inhalation of smoke from other people’s cigarettes is known as passive smoking^17^.

### Data collection

The electronic questionnaire was distributed to the 14239 participants, with data collectors available to assist as needed. The questionnaire was divided into several sections: the first covered sociodemographic details, including age, gender, marital status, education level, employment, and health status. The second section focused on behavioral factors, such as cigarette smoking, alcohol use, physical activity levels, and fast-food consumption. In the third section, participants reported on chronic health conditions, including chronic obstructive pulmonary disease, diabetes, cardiovascular conditions, hypertension, and obesity. Before beginning the survey, data collectors informed eligible patients about the study’s purpose and obtained their written consent. Participation was voluntary, with only those who consented proceeding to complete the questionnaire.

### Statistical analysis

Frequencies and percentages were calculated for categorical variables, including gender, marital status, employment status, education level, and general health status. For continuous variables, such as age, means and standard deviations are reported after confirming normality through histogram analysis. To examine associations between independent variables and the outcome (active and passive smoking), a multiple logistic regression analysis was conducted, with smoking status (active or passive, as a binary outcome) regressed on sociodemographic factors, household size, and current health status. Statistical significance was set at a p<0.05, and results included adjusted odds ratios (AORs) with 95% confidence intervals (CIs). All data were analyzed using SPSS version 26.0. Multicollinearity was performed within each of the multiple logistic regression analysis reported to ensure highly correlated variables did not distort the results.

## RESULTS

### Sociodemographic profile of the study population

The mean age of the population sample was 59.7 (SD=16.6) years. Table 1 presents the sociodemographic profile data for the population sample.

**Table 1:** Sociodemographic profile of the study sample of Riyadh (N=14239)

*Characteristics*	*n (%)*
**Age** (years)	
18–29	206 (1.4)
30–39	892 (6.3)
40–49	3172 (22.3)
50–59	3103 (21.8)
60–69	2533 (17.8)
70–79	1955 (13.7)
80–89	1228 (8.6)
90–100	675 (4.7)
**Gender**	
Male	6177 (43.4)
Female	8062 (56.6)
**Marital status**	
Not married	4939 (34.7)
Married	9300 (65.3)
**Employment status**	
Employed full-time	5942 (41.7)
Employed part-time	1375 (9.7)
Homemaker	2895 (20.3)
Retired	889 (6.2)
Student	1924 (13.5)
Unemployed due to illness/disability	299 (2.1)
Unemployed less than a year	409 (2.9)
Unemployed more than a year	506 (3.6)
**Education level**	
Primary school	572 (4.0)
Did not finish high school	669 (4.7)
High school	3268 (23.0)
Diploma	981 (6.9)
College graduate	6541 (45.9)
Master’s degree	599 (4.2)
Doctorate	196 (1.4)
**Health status**	
Excellent	4798 (33.7)
Very good	5076 (35.6)
Good	2815 (19.8)
Fair	1256 (8.8)
Poor	294 (2.1)

### Active smoking

Table 2 illustrates frequencies of active smoking. There were 17.3% of adults who reported smoking ‘almost always’ and 30.7% who were active smokers, however, said ‘not always’. Another 10.4% said they actively smoke ‘sometimes’. Sixty percent (60%) of active smokers were aware that their habitual active smoking is a serious problem. Another 15.2% indicated that it is a moderate problem.

**Table 2:** Prevalence and perception of active smoking in the study sample of Riyadh (N=14239)

	*n*	*%*
**Active smoking**		
Almost always	2459	17.3
Not always	4377	30.7
Never	5920	41.6
Sometimes	1483	10.4
**Perception of active smoking**		
Moderate problem	2160	15.2
Not a problem	1868	13.1
Not sure	1689	11.9
Serious problem	8522	59.8

### Passive smoking

The prevalence of passive smoking (secondhand smoke; environmental tobacco smoke/ETS) was 29% (with people reporting such exposure as ‘almost always’ or ‘sometimes’) as illustrated in Table 3. Being a serious problem regarding passive smoking, was reported by 58.2% of the sample, apart from another 14.6% who indicated passive smoking as a moderate problem causing adverse health outcomes.

**Table 3:** Passive smoking exposure and problem in the study sample of Riyadh (N=14239)

	*n*	*%*
**Passive smoking**		
Almost always	2344	16.5
Not always	4391	30.8
Never	5728	40.2
Sometimes	1776	12.5
**Passive smoking as a problem**		
Moderate problem	2077	14.6
Not a problem	2057	14.4
Not sure	1815	12.7
Serious problem	8290	58.2

### Multiple logistic regression analyses

*Active smoking behavior*

The multivariate logistic regression analysis identified several factors significantly associated with active smoking behavior (Table 4). Males were more likely to engage in active smoking compared to females, with an adjusted odds ratio (AOR) of 2.59 (95% CI: 2.23–3.02). Among education levels, individuals with a diploma demonstrated increased odds of smoking (AOR=1.64; 95% CI: 1.37–1.95), while other educational categories did not show significant associations. Household size also emerged as a significant factor, with individuals living in households of 3–5 members (AOR=1.48; 95% CI: 1.22–1.79) and those in households of ≥6 members (AOR=2.26; 95% CI: 1.40–3.65) having higher odds of smoking compared to smaller households. Employment status was another significant determinant, with students exhibiting the highest likelihood of smoking (AOR=3.71; 95% CI: 2.36–5.81), followed by individuals unemployed for less than a year (AOR=2.01; 95% CI: 1.35–3.00). Annual household income showed an inverse relationship with smoking behavior, particularly in individuals earning 50000–59999 SAR, who had markedly reduced odds of smoking (AOR=0.29; 95% CI: 0.22–0.38). Perceived health status was strongly associated with smoking, with those reporting good health showing the highest odds (AOR=2.96; 95% CI: 1.68–5.25), followed by those reporting excellent health (AOR=2.41; 95% CI: 1.86–3.12). These results highlight the complex interplay of sociodemographic, economic, and health-related factors influencing active smoking behavior.

**Table 4:** Multivariate logistic regression analysis of factors associated with active smoking behavior (N=14239)

*Variables*	*p*	*AOR*	*95% CI*	
			*Lower*	*Upper*
**Age** (years)	0.01	0.99	0.98	0.99
**Gender** (male)	<0.001	2.59	2.23	3.02
**Marital status** (married)	0.55	0.95	0.79	1.13
**Education level**	<0.001			
College graduate	0.45	1.27	0.67	2.39
Doctorate	0.68	0.93	0.66	1.31
Did not finish high school	0.69	1.05	0.83	1.32
Diploma	<0.001	1.64	1.37	1.95
High school	0.08	1.36	0.96	1.92
Master’s degree	0.06	1.29	0.99	1.68
Other	0.31	1.20	0.85	1.71
Primary school	0.31	1.20	0.85	1.71
**Number of people living in the household**	<0.001			
1–2	0.04	1.19	1.00	1.43
3–5	<0.001	1.48	1.23	1.79
≥6	<0.001	2.26	1.40	3.65
**Employment status**	<0.001			
Employed full-time	0.55	0.93	0.74	1.18
Employed part-time	0.79	0.97	0.79	1.19
Homemaker	0.29	0.83	0.59	1.17
Retired	0.31	0.88	0.68	1.13
Student	<0.001	3.71	2.39	5.81
Unemployed due to illness or disability	0.083	1.45	0.95	2.21
Unemployed less than a year	<0.001	2.01	1.34	3.00
**Annual household income** (SAR)	<0.001			
10000–19000	<0.001	0.55	0.41	0.75
100000–149999	<0.001	0.47	0.34	0.65
≥150000	0.12	0.81	0.62	1.06
20000–29999	0.10	1.31	0.95	1.80
30000–39999	0.99	0.99	0.71	1.40
40000–49999	0.47	0.87	0.61	1.26
50000–59999	<0.001	0.29	0.22	0.38
60000–69999	0.66	0.91	0.62	1.35
70000–79999	<0.001	0.50	0.33	0.75
80000–89999	0.001	0.57	0.39	0.84
90000–99000	<0.001	1.52	1.25	1.83
**Current health status**	<0.001			
Excellent	0.001	2.41	1.86	3.12
Fair	0.001	1.31	1.10	1.56
Good	<0.001	2.96	1.67	5.25
Poor	<0.001	1.37	1.16	1.62
All other categories compared to poor status	<0.001	2.97	1.67	5.25

AOR: adjusted odds ratio. SAR: 1000 Saudi Riyals about US$270.

*Passive smoking behavior*

The multivariate logistic regression analysis for passive smoking behavior identified several significant associations as illustrated in Table 5. Males were more likely to experience passive smoking than females, with an adjusted odds ratio (AOR) of 2.59 (95% CI: 2.23–3.02). Education level also played a role, with individuals holding a diploma (AOR=1.47; 95% CI: 1.22–1.75) and those with a Master’s degree (AOR=1.37; 95% CI: 1.04–1.80) being more likely to engage in passive smoking, while other education levels were not significantly associated.

**Table 5:** Multivariate logistic regression analysis of factors associated with passive smoking behavior (N=14239)

*Variables*	*p*	*AOR*	*95% CI*	
			*Lower*	*Upper*
**Age** (years)	0.01	0.99	0.98	1.01
**Gender** (male)	<0.001	2.60	2.23	3.02
**Marital status** (married)	0.55	0.95	0.80	1.13
**Education level**	<0.001			
College graduate	0.88	0.95	0.51	1.78
Doctorate	0.68	0.93	0.66	1.31
Did not finish high school	0.61	1.06	0.84	1.35
Diploma	<0.001	1.47	1.26	1.75
High school	0.19	1.27	0.89	1.79
Master’s degree	0.02	1.37	1.05	1.80
Other	0.08	1.39	0.97	2.00
Primary school	<0.001			
**Number of people living in the household**	0.022	1.23	0.03	1.47
1–2	<0.001	1.57	1.30	1.91
3–5	0.001	2.27	1.39	3.72
≥6	<0.001			
**Employment status**	0.88	0.98	0.77	1.25
Employed full-time	0.70	0.96	0.79	1.18
Employed part-time	0.32	0.84	0.59	1.19
Homemaker	0.66	0.95	0.74	1.21
Retired	<0.001	4.00	2.45	6.52
Student	0.01	1.89	1.20	2.97
Unemployed due to illness or disability	0.003	1.85	1.23	2.78
Unemployed less than a year	<0.001			
**Annual household income** (SAR)	<0.001	0.42	0.31	0.57
10000–19000	<0.001	0.34	0.24	0.48
100000–149999	0.052	0.76	0.58	1.00
≥150000	0.85	1.03	0.74	1.44
20000–29999	0.11	0.76	0.54	1.07
30000–39999	0.028	0.66	0.46	0.96
40000–49999	<0.001	0.32	0.25	0.42
50000–59999	0.042	0.66	0.45	0.99
60000–69999	<0.001	0.48	0.33	0.73
70000–79999	<0.001	0.43	0.29	0.63
80000–89999	0.091	1.19	0.97	1.5
90000–99000	<0.001			
<100000	<0.001	2.13	1.64	2.78
**Current health status**	<0.001	1.43	1.20	1.71
Excellent	<0.001	3.60	1.93	6.70
Fair	<0.001	1.44	1.22	1.70
Good	0.88	0.95	0.51	1.78
Poor	0.68	0.93	0.66	1.31
All other categories compared to poor status	0.61	1.06	0.84	1.35

AOR: adjusted odds ratio. SAR: 1000 Saudi Riyals about US$270.

Household size showed a strong positive association, with individuals in households of 3–5 members having higher odds of passive smoking (AOR=2.27; 95% CI: 1.38–3.72), followed by those in households of 1–2 members (AOR=1.57; 95% CI: 1.29–1.91). Employment status was also influential, as retired individuals had a significantly increased likelihood of passive smoking (AOR=1.85; 95% CI: 1.23–2.78), followed by those unemployed due to illness or disability (AOR: 1.85; 95% CI: 1.23–2.78) and students (AOR=1.88; 95% CI: 1.20–2.96).

Annual household income showed a notable inverse relationship, with individuals earning 40000–49999 SAR (AOR=0.32; 95% CI: 0.24–0.42) and those in the 10000–19000 SAR bracket (AOR=0.33; 95% CI: 0.24–0.46) having the lowest odds of passive smoking. Perceived health status was significantly associated, as individuals reporting excellent health had the highest odds of passive smoking (AOR=3.59; 95% CI: 1.93–6.69), followed by those reporting fair health (AOR=1.44: 95% CI: 1.22–1.69). These findings highlight how sociodemographic, economic, and health factors collectively influence passive smoking behavior .

## DISCUSSION

This cross-sectional study examined the prevalence, perceptions, and predictors of active and passive smoking among adults visiting primary healthcare centers in Riyadh, Saudi Arabia. The findings provide critical insights into the burden of tobacco exposure and highlight several sociodemographic and health-related factors associated with smoking behaviors.

The prevalence of active smoking in the study population revealed that 17.3% of participants reported smoking ‘almost always’, while 30.7% smoked ‘not always’. These findings align with national estimates in Saudi Arabia, where the prevalence of tobacco use has been reported to be between 17% and 24% in similar age groups, reflecting the persistent burden of smoking in the region^3^. Globally, these figures are lower than those reported in regions with high tobacco use, such as South-East Asia and parts of Europe, but they underscore the importance of targeted interventions, given the increasing trend of smoking among young adults in Middle Eastern countries^18^.

The perception of smoking as a serious problem was high, with nearly 60% of participants identifying it as such. This level of concern suggests growing awareness about the health risks of smoking, which may result from public health campaigns and anti-smoking policies implemented in Saudi Arabia, such as taxation on tobacco products and public smoking bans^19^. However, 13.1% of participants did not perceive smoking as a problem, and 11.9% were unsure, indicating that misperceptions about tobacco’s health risks still exist in certain sections of the population. Previous studies in Saudi Arabia have highlighted similar disparities in risk perception, particularly among individuals with a lower level of education^20^.

Regarding passive smoking, 16.5% of participants reported being exposed to secondhand smoke (SHS) ‘almost always’, while 30.8% experienced it ‘not always’. The prevalence of SHS exposure in this study is comparable to findings from neighboring Gulf Cooperation Council (GCC) countries, where exposure rates vary across countries^21^. For example, the overall exposure to SHS was notably high, with adolescents in Oman experiencing SHS at home and school ranging from 12.7% to 20.7%, while in Kuwait, these figures increased to 39.4% and 36.7%, respectively. Additionally, nearly half of the adolescents reported SHS exposure in public places, with prevalence rates ranging from 40.8% in Saudi Arabia to as high as 65.9% in Kuwait^21^. While SHS exposure remains a public health concern worldwide, its prevalence in this study is considerably higher than in many Western nations, where stricter enforcement of smoking bans in homes and public spaces has reduced exposure rates^22^. Furthermore, notably, 58.2% of participants perceived SHS as a serious problem, but 14.4% did not consider it a problem, indicating a gap in public awareness about the dangers of environmental tobacco smoke (ETS). This gap mirrors findings from prior research, which identified limited knowledge about SHS hazards, particularly among non-smokers in Middle Eastern contexts.

The findings from the current study offer important insights into the predictors of active and passive smoking in the Saudi Arabian population, reflecting regional smoking behaviors and exposure patterns while also highlighting differences from global trends. Active smoking was strongly associated with male gender, specific educational levels (such as holding a diploma), and larger household sizes. These results align with other studies in the Gulf Cooperation Council (GCC) region, which have consistently shown higher smoking prevalence among men due to cultural and social acceptability of smoking among males^14^. However, the association between education level and smoking varies globally. While higher levels of education typically reduce smoking prevalence in Western countries, the association seen in this study supports findings from neighboring Middle Eastern nations, where smoking is sometimes viewed as a marker of social status among educated youth^23,24^. Globally, predictors of smoking behaviors often differ significantly from those observed in this study. For instance, in Western countries, female smoking prevalence is higher compared to the stark gender disparity seen in Saudi Arabia^25^. This contrast reflects cultural and societal norms that discourage smoking among women in Middle Eastern societies. Another important distinction lies in the relationship between education and smoking. Globally, higher levels of education are typically associated with lower smoking prevalence due to greater health awareness^26^. However, in this study and other regional research, the opposite trend is evident, highlighting the sociocultural contexts that influence tobacco use patterns in Middle Eastern populations.

The findings regarding the relationship between education level and smoking behaviors in this study align with some patterns reported in Middle Eastern populations but diverge from global trends. In Saudi Arabia and the broader Middle East and North Africa (MENA) region, higher levels of education, particularly holding a diploma or Master’s degree, have been associated with increased smoking prevalence in certain groups, contrasting with Western countries where higher levels of education generally corresponds to lower smoking rates due to greater health awareness and stricter tobacco regulation^23,24^. However, some studies reported no association between education level and smoking behaviors^20^.

This divergence may be attributed to sociocultural factors specific to the MENA region. For example, smoking is often seen as a social norm or even a status symbol among educated males, particularly in urban areas where smoking behaviors can be associated with social gatherings or professional settings^27^. In contrast, in countries like Lebanon and Jordan, smoking prevalence remains higher among the less educated, reflecting the complex interplay of socioeconomic and cultural influence^23,24^. Likewise, socio-cultural and environmental factors contributing to female adolescents’ smoking tendency, including parenting patterns, family modeling, community predisposition, media negative impact, peer role modeling, and group membership^28^.

The relationship between household size and both active and passive smoking behaviors is well-documented in studies across the GCC countries, with larger households often experiencing higher exposure to secondhand smoke (SHS)^29^. This association can be partly explained by cultural practices that encourage extended families to live together, where smoking indoors remains a social norm. In these environments, the presence of active smokers increases the likelihood of SHS exposure, which is particularly concerning given the adverse health outcomes associated with secondhand smoke. Furthermore, the family-oriented culture in many GCC countries, such as Saudi Arabia, often leads to shared living spaces where the risks of SHS exposure are elevated^29^.

Research across the MENA region supports these findings, highlighting the persistence of SHS exposure in household settings despite efforts to curtail tobacco use in public spaces. In some GCC countries, smoking bans in public areas have not fully addressed the domestic sources of SHS exposure, particularly in larger households, which are more likely to contain smokers^27^. These findings suggest that social dynamics, combined with limited enforcement of smoke-free regulations at home, play a significant role in continuing high levels of SHS exposure.

The role of income also warrants attention. Lower household income was associated with higher smoking behaviors and SHS exposure. This trend is consistent with global literature, which often reports higher smoking prevalence among economically disadvantaged groups^30^. In contrast, wealthier populations tend to have better access to smoking cessation programs and greater awareness of tobacco’s health risks. However, the income disparities observed in this study are particularly relevant to Saudi Arabia, where rapid socioeconomic changes and urbanization have influenced tobacco consumption patterns^31^. The association between lower socioeconomic status (SES) and higher smoking prevalence is well-documented in global literature^32^. Studies consistently show that economically disadvantaged groups, including those with lower income and lower levels of education, are more likely to smoke^32,33^. This trend is particularly evident in both high-income and middle-income countries, where tobacco consumption is often higher in these populations due to factors such as affordability of tobacco products, limited access to cessation resources, and social norms that may condone smoking^32^. In the United States, for example, research has demonstrated that individuals facing cumulative socioeconomic disadvantages – such as low income, limited education, and unemployment – are significantly more likely to smoke and less likely to quit smoking compared to more advantaged groups^32^. This pattern is also seen in other regions, including the MENA, where smoking remains prevalent among lower income groups^33^. Tobacco use in such populations is often influenced by factors such as targeted advertising, the lower cost of tobacco, and social dynamics that may normalize smoking as part of daily life^27^. Moreover, smoking prevalence in these populations may be compounded by a lack of education and public health awareness, which are also critical factors influencing smoking behaviours^27,33^.

Notably, perceptions of health status also influenced both active and passive smoking behaviors. Individuals reporting better health were paradoxically more likely to smoke or be exposed to SHS. This finding is consistent with regional studies which suggest many smokers in the Middle East underestimate the health risks associated with tobacco use, potentially due to limited awareness or cultural normalization of smoking. The finding is consistent with regional studies suggesting that many smokers in this region may underestimate the health risks associated with tobacco use^34,35^. This can be attributed to limited awareness about the harmful effects of smoking and cultural normalization of tobacco use, particularly in social contexts^34,35^. The prevalence of tobacco use, including smoking and SHS exposure, is often higher in environments where smoking is socially accepted or even encouraged, despite its well-documented health risks.

### Strengths and limitations

This study has several strengths. The use of a large, nationally representative sample from Riyadh, Saudi Arabia, enhances the generalizability of the findings to a wide range of individuals attending primary healthcare centers. The application of multivariate logistic regression allows for the identification of sociodemographic and health-related predictors of active and passive smoking, offering a more nuanced understanding of the factors influencing smoking behaviors. Additionally, the use of a comprehensive electronic questionnaire facilitates the collection of standardized and reliable data.

However, there are also notable limitations. First, self-reported data can introduce social desirability bias, where participants may provide responses that they believe are socially acceptable rather than truthful. This can skew the results and limit the validity of the findings. Additionally, the crosssectional design used in the study means that only a snapshot of data is collected at one point in time, making it difficult to establish causal relationships between variables. Second, self-reported data on smoking behaviors, while common in epidemiological studies, may be subject to recall or social desirability biases, particularly in relation to sensitive behaviors such as smoking. Third, the study’s focus on Riyadh may limit the external validity of the findings to other regions in Saudi Arabia, where cultural and socioeconomic factors may differ. Fourth, in our study, we acknowledge the potential health-related bias in our sample, as individuals visiting healthcare centers may have worse health conditions compared to the general population. This bias might lead to an overestimation of active and passive smoking rates among less healthy populations. It is important to consider this when interpreting the results and discussing the generalizability of our findings. Also, our sample may not be entirely representative of the overall population. Lastly, the study did not assess the impact of tobacco control policies, which may influence smoking behavior, making it difficult to draw conclusions about the effects of public health interventions.

### Implications

Saudi Arabia, a Party to the World Health Organization Framework Convention on Tobacco Control, passed Royal Decree No. 56 in 2015 to combat tobacco use. The law focuses on smoke-free places, advertising, promotion, sponsorship, packaging, and labeling. Since 2016, smoking has been banned in various settings, including mosques, ministries, factories, educational institutions, workplaces, and public transport. The regulation also prohibits selling cigarettes at smaller retailers and tobacco product displays at the point of sale. However, evidence on compliance and visibility of cigarette retailers around educational facilities is lacking. The World Health Organization recommends raising tobacco excise taxes to account for at least 70% of retail prices^19^.

According to the findings of the study there is a need to intensify public health campaigns, stricter enforcement of smoking bans in public and private spaces, and targeted interventions for high-risk groups. Educational programs should focus on correcting misconceptions about SHS and active smoking risks, particularly among men, students, and households with higher exposure rates. Future research should explore the underlying sociocultural factors that perpetuate smoking norms and assess the long-term impact of current tobacco control measures in reducing smoking prevalence in Saudi Arabia.

## CONCLUSIONS

The study’s findings underscore the complex interplay of sociodemographic, economic, and health-related factors influencing active and passive smoking in Saudi Arabia. While some results align with global trends, others reflect unique regional dynamics, including cultural norms, socioeconomic disparities, and misconceptions about tobacco risks. These findings highlight the need for culturally tailored public health interventions that address specific predictors of smoking behaviors in Saudi Arabia. Efforts should focus on reducing SHS exposure through stricter enforcement of smoking bans, targeted educational campaigns to improve health awareness, and initiatives to reduce smoking prevalence in high-risk groups such as men and large households. Future research should further explore the sociocultural determinants of smoking and evaluate the effectiveness of current tobacco control measures in the region.

## Data Availability

The datasets supporting the conclusions of this article are included within the article.

## References

[1] World Health Organization. Tobacco. WHO; 2023. Accessed February 25, 2025. https://www.who.int/en/news-room/fact-sheets/detail/tobacco

[2] Jiang C, Chen Q, Xie M. Smoking increases the risk of infectious diseases: a narrative review. Tob Induc Dis. 2020;18:60. doi:10.18332/tid/123845 32765200 PMC7398598

[3] Moradi-Lakeh M, El Bcheraoui C, Tuffaha M, et al. Tobacco consumption in the Kingdom of Saudi Arabia, 2013: findings from a national survey. BMC Public Health. 2015;15:611. doi:10.1186/s12889-015-1902-3 26141062 PMC4491232

[4] Algabbani AM, Almubark RA, Althumiri NA, Alqahtani AS, BinDhim NF. The prevalence of cigarette smoking in Saudi Arabia in 2018. Food Drug Regul Sci J. 2018. doi:10.32868/rsj.v1i1.22

[5] Amin TT, Amr MA, Zaza BO. Psychosocial predictors of smoking among secondary school students in Al-Hassa, Saudi Arabia. J Behav Med. 2011;34(5):339-350. doi:10.1007/s10865-011-9319-7 21286799

[6] So ES, Yeo JY. Factors associated with early smoking initiation among Korean adolescents. Asian Nurs Res (Korean Soc Nurs Sci). 2015;9(2):115-119. doi:10.1016/j.anr.2015.05.002 26160239

[7] Gaffar AM, Alsanosy RM, Mahfouz MS. Sociodemographic factors associated with tobacco smoking among intermediate and secondary school students in Jazan Region of Saudi Arabia. Subst Abus. 2013;34(4):381-388. doi:10.1080/08897077.2013.779361 24159909 PMC3827661

[8] Fida HR, Abdelmoneim I. Prevalence of smoking among secondary school male students in Jeddah, Saudi Arabia: a survey study. BMC Public Health. 2013;13:1010. doi:10.1186/1471-2458-13-1010 24160571 PMC3840679

[9] Kristjansson AL, Sigfusdottir ID, Allegrante JP, Helgason AR. Social correlates of cigarette smoking among Icelandic adolescents: a population-based cross-sectional study. BMC Public Health. 2008;8:86. doi:10.1186/1471-2458-8-86 18328089 PMC2322976

[10] Margolis R. Educational differences in healthy behavior changes and adherence among middle-aged Americans. J Health Soc Behav. 2013;54(3):353-368. doi:10.1177/0022146513489312 23988727 PMC3998203

[11] Bourassa KJ, Ruiz JM, Sbarra DA. Smoking and physical activity explain the increased mortality risk following marital separation and divorce: evidence from the English longitudinal study of ageing. Ann Behav Med. 2019;53(3):255-266. doi:10.1093/abm/kay038 29796660 PMC6374715

[12] Phillips E, Wang TW, Husten CG, et al. Tobacco product use among adults - United States, 2015. MMWR Morb Mortal Wkly Rep. 2017;66(44):1209-1215. doi:10.15585/mmwr.mm6644a2 29121001 PMC5679591

[13] Issrani R, Alruwaili DSR, Alruwaili RHG, et al. Patterns and associated factors of shisha usage among the undergraduate students of Jouf University, Saudi Arabia: a cross-sectional study. Tob Induc Dis. 2024;22:10.18332/tid/186185. doi:10.18332/tid/186185 PMC11131362 38807709

[14] Monshi SS, Arbaein TJ, Alzhrani AA, et al. Factors associated with the desire to quit tobacco smoking in Saudi Arabia: evidence from the 2019 Global Adult Tobacco Survey. Tob Induc Dis. 2023;21:33. doi:10.18332/tid/159735 36875735 PMC9983308

[15] Alnashri AMM, Alshehri AAA, AlQarni SND, et al. Association between tobacco smoking and different demographic factors among the general population at Al-Baha, Saudi Arabia. Popul Med. 2024;6(November):32. doi:10.18332/popmed/199563

[16] Sinha SK, Haider MN. Tobacco and health effects. In: Sinha SK, Pal R, Ghosh A, Rana RK, eds. Tobacco & Public Health: Tobacco and Community Well-being – A Worldwide Health Crisis. Shashwat Publication; 2024.

[17] Naseeb U, Alam MT, Pervez F, et al. Knowledge, attitude, and perception of passive smoking among medical and dental students of Karachi: a survey-based study. Tob Use Insights. 2024;17:1179173X241258347. doi:10.1177/1179173X241258347 PMC11119405 38800830

[18] World Health Organization. Tobacco use declines despite tobacco industry efforts to jeopardize progress. WHO; 2024. Accessed February 25, 2025. https://www.who.int/news/item/16-01-2024-tobacco-use-declines-despite-tobacco-industry-efforts-to-jeopardize-progress

[19] AlJishi H, Kusuma D, AlQurashi A, AlFaiz A, AlSaad A, Aljishi M. Compliance with tobacco control policy and visibility of cigarette retailers around educational facilities in Riyadh, Saudi Arabia. Front Public Health. 2022;10:713460. doi:10.3389/fpubh.2022.713460 35719605 PMC9198959

[20] Mujoo S, Alqahtani AS, Hamdi BA, et al. Knowledge and perception about health risks of cigarette smoking among youngsters in Jazan Region, Saudi Arabia. J Pharm Bioallied Sci. 2024;16(Suppl 1):S308-S313. doi:10.4103/jpbs.jpbs_503_23 38595467 PMC11001078

[21] Al-Zalabani AH. Secondhand smoke exposure among adolescents in the Gulf Cooperation Council countries: analysis of Global Youth Tobacco Surveys. Sci Rep. 2024;14:21534. doi:10.1038/s41598-024-72314-1 39278959 PMC11402983

[22] Ma C, Heiland EG, Li Z, Zhao M, Liang Y, Xi B. Global trends in the prevalence of secondhand smoke exposure among adolescents aged 12-16 years from 1999 to 2018: an analysis of repeated cross-sectional surveys. Lancet Glob Health. 2021;9(12):e1667-e1678. doi:10.1016/S2214-109X(21)00365-X 34571047

[23] Abdulrahim S, Jawad M. Socioeconomic differences in smoking in Jordan, Lebanon, Syria, and Palestine: a cross-sectional analysis of national surveys. PLoS One. 2018;13(1):e0189829. doi:10.1371/journal.pone.0189829 29381734 PMC5790213

[24] Sibai AM, Iskandarani M, Darzi A, et al. Cigarette smoking in a Middle Eastern country and its association with hospitalisation use: a nationwide cross-sectional study. BMJ Open. 2016;6(4):e009881. doi:10.1136/bmjopen-2015-009881 PMC4838686 27059466

[25] Jafari A, Rajabi A, Gholian-Aval M, Peyman N, Mahdizadeh M, Tehrani H. National, regional, and global prevalence of cigarette smoking among women/females in the general population: a systematic review and meta-analysis. Environ Health Prev Med. 2021;26(1):5. doi:10.1186/s12199-020-00924-y 33419408 PMC7796590

[26] Taylor RS. Using statistical modelling approaches to explore the prevalence of cigarette smoking in the Global South. Dissertation. University of Southampton; 2024.

[27] Sultan Y, Salman Z, Alzaatreh M, et al. Smoking-related disease impact in the Eastern Mediterranean region: a comprehensive assessment using global burden of disease data. Asian Pac J Cancer Prev. 2024;25(2):495-505. doi:10.31557/APJCP.2024.25.2.495 38415535 PMC11077135

[28] Jafari A, Mahdizadeh M, Peyman N, Gholian-Aval M, Tehrani H. Exploration the role of social, cultural and environmental factors in tendency of female adolescents to smoking based on the qualitative content analysis. BMC Womens Health. 2022;22(1):38. doi:10.1186/s12905-022-01617-0 35148756 PMC8832822

[29] Koronaiou K, Al-Lawati JA, Sayed M, et al. Economic cost of smoking and secondhand smoke exposure in the Gulf Cooperation Council countries. Tob Control. 2021;30(6):680-686. doi:10.1136/tobaccocontrol-2020-055715 32817575 PMC8543216

[30] Hiscock R, Bauld L, Amos A, Fidler JA, Munafò M. Socioeconomic status and smoking: a review. Ann N Y Acad Sci. 2012;1248:107-123. doi:10.1111/j.1749-6632.2011.06202.x 22092035

[31] Alamoudi WA, Alsobhi WK, Dhelemi AA, et al. Smoking prevalence and associated factors among the general population living in the Makkah Region, Saudi Arabia. Saudi J Intern Med. 2024;13(1):21-27. doi:10.4103/SJIM.SJIM_3_24

[32] Leventhal AM, Bello MS, Galstyan E, Higgins ST, BarringtonTrimis JL. Association of cumulative socioeconomic and health-related disadvantage with disparities in smoking prevalence in the United States, 2008 to 2017. JAMA Intern Med. 2019;179(6):777-785. doi:10.1001/jamainternmed.2019.0192 31009023 PMC6547249

[33] Casetta B, Videla AJ, Bardach A, et al. Association between cigarette smoking prevalence and income level: a systematic review and meta-analysis. Nicotine Tob Res. 2017;19(12):1401-1407. doi:10.1093/ntr/ntw266 27679607

[34] Vupputuri S, Hajat C, Al-Houqani M, et al. Midwakh/dokha tobacco use in the Middle East: much to learn. Tob Control. 2016;25(2):236-241. doi:10.1136/tobaccocontrol-2013-051530 25342581 PMC4789808

[35] Abu-Rmeileh NME, Khader YS, Abdul Rahim H, et al. Tobacco control in the Eastern Mediterranean region: implementation progress and persisting challenges. Tob Control. 2022;31(2):150-152. doi:10.1136/tobaccocontrol-2021-056539 35241580

